# Device-Based Movement Behaviors, Executive Function, and Academic Skills among African American Children with ADHD and Disruptive Behavior Disorders

**DOI:** 10.3390/ijerph19074032

**Published:** 2022-03-29

**Authors:** María Enid Santiago-Rodríguez, Jared D. Ramer, David X. Marquez, Stacy L. Frazier, Catherine L. Davis, Eduardo E. Bustamante

**Affiliations:** 1 School of Kinesiology, University of Michigan, Ann Arbor, MI 48109, USA; marenid@umich.edu; 2 Department of Kinesiology & Nutrition, University of Illinois Chicago, Chicago, IL 60612, USA; jramer2@uic.edu (J.D.R.); marquezd@uic.edu (D.X.M.); 3 College of Arts, Sciences & Education, Florida International University, Miami, FL 33199, USA; slfrazi@fiu.edu; 4 Department of Medicine, Georgia Prevention Institute, Augusta University, Augusta, GA 30912, USA; katie.davis@augusta.edu

**Keywords:** poverty, physical activity, accelerometry, sedentary time, health disparities, social determinants of health

## Abstract

Background: Physical activity (PA) has been identified as a promising intervention to improve executive function (EF) and reduce ADHD symptoms in children. Few African American children with ADHD and Disruptive Behavior Disorders (DBDs) from families with low incomes are represented in this literature. The purpose of this study is to test the relationships between PA and sedentary time (ST), and EF and academic skills among African American children with ADHD and DBD from low-income families. Methods: Children (*n* = 23, 6–13 years old) wore an ActiGraph for one week to measure PA and ST. EF was measured through parent report and direct neuropsychological tests. Academic skills were measured with the Curriculum-Based Measurement System. Bivariate correlations tested relationships between PA, ST, EF, and academic skills. Results: A significant correlation was observed between vigorous PA time and parent reported EF (*r* = −0.46, *p* = 0.040). Light PA and moderate PA were not related to EF or academic skills, and neither was ST. Conclusions: Vigorous PA may prove useful as an adjunct treatment to improve EF in African American children with ADHD and DBD in low-income neighborhoods. Research using experimental and longitudinal designs, and examining qualitative features of PA experiences, will be critical for understanding relationships between PA, academic skills, and EF in this population.

## 1. Introduction

Results from intervention studies and meta-analyses suggest that physical activity (PA) improves executive function (EF) [1] and reduces symptoms among children with Attention-Deficit Hyperactivity/Impulsivity Disorder (ADHD) [2,3] and that PA in earlier life stages predicts symptom severity in later life stages [4,5]. These findings hold true across measurements of ADHD symptoms and EF [6], including parent and teacher reports, neuropsychological tests of EF [7], and classroom observations [8], with age ranges from middle childhood to early adulthood [4]. Additionally, improving EF through PA has the potential to reduce symptoms each day following PA bouts [9] and over the course of development [10]. In turn, these reductions in symptoms could improve classroom engagement, which could improve content comprehension and test performance [11]. A similar but inverse set of relationships is believed to occur with sedentary time (ST). More ST, especially recreational screen time, is thought to exacerbate cognitive deficits [12], ADHD symptoms, and poor academic performance [13]. Thus, associations are expected between PA and ST, and cognitive and academic outcomes among children with ADHD. This evidence is so sufficiently strong that, in the United Kingdom, advising families of children with ADHD to adopt active lifestyles has become part of treatment guidelines for clinicians [14].

Nevertheless, important gaps in knowledge remain. Most notably, few studies have included participants from racial/ethnic minority backgrounds, living in low-income communities, or the combination of the two (i.e., children with ADHD from racial/ethnic minority backgrounds also living in low-income communities). Cross-sectional studies and intervention trials supporting that PA improves EF and reduces symptoms have largely utilized samples from middle-high socioeconomic status communities. For instance, a cross-sectional study demonstrating relationships between EF and movement behaviors reported that 24% of their participants were from families with an annual income between USD 75,000 and 99,999 and 47% of the participants were from families with an annual income > USD 100,000 [15]. Similarly, a successful trial reported that the average socioeconomic status of participants was six points on a scale of zero to eight, in which higher numbers corresponded with higher socioeconomic status [16]. Moreover, longitudinal studies and cross-sectional studies have acknowledged the lack of racial/ethnic diversity in the literature as a major limitation, e.g., [4,15]. 

Much of the extant literature has focused on how neurobiological pathways may explain the benefits of PA programs for reducing ADHD symptoms. For instance, it has been proposed that neurotransmitter regulation is a potential pathway for explaining improvement. This is because neurotransmitters have an important role in ADHD pathophysiology [17]; the prefrontal cortex and dopaminergic systems act as a network when a person is engaged in cognitive tasks or trying to achieve behavioral control [18]. These systems are affected by ADHD medication and are also affected by exercise [19]. Thus, it is reasonable to surmise that if children with ADHD receive a neurotransmitter regulation benefit from PA, then real-world behavior should benefit, regardless of background or demographics. However, for systemically marginalized and racial/ethnic minoritized children with ADHD, social determinants of health serve to influence ADHD outcomes through multiple pathways (e.g., stigma, stress, chaotic environments, perceived discrimination), minimize access to health promoting resources (e.g., housing, nutrient-rich foods, green space, health care, education), and increase barriers to academic achievement [20]. Hence, while PA may be sufficient to improve real-world behavior and academic performance in higher income majority children via its neurobiological benefits, this may be insufficient to influence real-world behavior in subpopulations. Research in this area is especially critical when one considers the high levels of unmet mental health need in racial/ethnic minority and low-income communities.

In the United States, African American children are less likely than children from other racial/ethnic backgrounds to receive ADHD treatment and to adhere to treatment when it is received [21]. Psychosocial treatments are the frontline treatment for ADHD, but it has been estimated that only one in five African American children meeting criteria for ADHD receives services [22]. Among these, half discontinue treatment prematurely [23]. Medication, the second line of treatment, is utilized by fewer than 10% of African American children with ADHD each year, and the average time to discontinuation has been estimated to be as low as four months [24]. African American parents are less likely to view ADHD as a medical condition requiring treatment [25], are more likely to have negative expectations of their child’s ADHD treatment [26] and are less likely to administer medication compared to White parents because of concerns about efficacy and side effects [27]. Finally, African American children with ADHD are 65% more likely than White children to present with comorbid ADHD and Oppositional Defiant Disorder [28]. Oppositional Defiant Disorder and Conduct Disorder are grouped together under Disruptive Behavior Disorders (DBD) because they share a trajectory of escalating hostile and defiant behaviors that violate age-appropriate norms and rules [29]. ADHD and DBD are highly comorbid: 26% of children with ADHD have DBD, and 67% with DBD have ADHD [28].

The purpose of this study was to test the relationships between device-based PA and ST, and EF and academic skills among African American children with ADHD and/or DBD from low-income families. It is hypothesized that (1) minutes of PA at moderate and vigorous intensities, but not light intensity PA, would be related to better EF and academic skills; and (2) minutes of ST would be related to poorer EF and academic skills. Findings from this study will provide information about the applicability of current knowledge to this understudied group.

## 2. Materials and Methods

### 2.1. Participants and Recruitment

The current study is a secondary data analysis of baseline data from a pilot randomized controlled trial of multi-week PA after-school for African American children with ADHD and DBD (R36 MH093152) [3,30]. The trial included 42 children who screened positive for ADHD and/or DBD, as well as 14 of their non-disruptive siblings to accommodate parent after-school supervision needs. Accelerometers were distributed to all 56 children at baseline. Of these, 23 children provided valid accelerometer data and are included in this analysis. Participants were African American children that attended the same school in a predominantly African American low-income neighborhood in an urban center. Most met positive or intermediate criteria for ADHD (65%), oppositional defiant disorder (48%), and conduct disorder (7%) according to the National Institute of Mental Health Diagnostic Interview Schedule for Children, Version IV, Parent Interview (NIMH DISC-IV-P). Four of the participants were non-disruptive siblings and did not meet criteria for any disorder. Sex distribution was even (males: 52.2%) and more than half of the children (57%) were classified as overweight or obese based on BMI percentile obtained by the CDC BMI Percentile Calculator for Child and Teen [31]. Children were between six and thirteen years old and the average age was 9.2 ± 1.91 years. All parents reported income below the poverty level in the United States and 65% of children were being raised by a single parent. 

Recruitment followed a detailed screening protocol described previously [3]. Briefly, children were included if they had a positive teacher and/or parent screen on the DBD Rating Scale [32], which was based on an endorsement of ≥6 items on the ADHD inattention scale, ≥6 items on the ADHD hyperactivity/impulsivity scale, ≥4 items on the oppositional defiant disorder scale, or ≥3 items on the conduct disorder scale. Additionally, a parent and/or teacher needed to report a positive screening in the Impairment Rating Scale [33] positive screen, which means rating ≥ 3 in any of the following domains: siblings, parents, peers, academics, self-esteem, and family. In addition, non-disruptive siblings of enrolled children were also eligible to participate and four provided data for this study. Following enrollment, we conducted the Diagnostic Interview Schedule for Children, version 4, parental interview (DISC-IV-P) [34]. This yielded either a positive (DSM-IV-P diagnosis criteria met), intermediate (symptomatology and impairments are present, but do not met diagnosis criteria), or negative (minimal symptomatology across diagnoses) diagnosis for each child. A clinical psychologist (S.L.F.) and the PI (E.E.B.) trained two graduate students to administer the DISC-IV-P. 

### 2.2. Procedures

This study was approved and overseen by the University of Illinois Chicago IRB as well as the Chicago Public Schools Research Review Board. Prior to data collection, parents provided in-person written informed consent and children provided written assent. Data for the current study were collected during winter 2013, which represents baseline measurements only. A trained research assistant conducted DISC-IV-P, EF, and academic skills measurements individually at baseline, in a private room. Accelerometers were provided to 56 children during the first two weeks of the program; 23 children returned the device with valid data and are included in analyses. 

### 2.3. Measurements

**Executive function** was measured through the Behavior Rating Inventory of Executive Function Global Executive Composite Score (BRIEF GEC T-score), which was completed by parents, and by the Automated Working Memory Assessment (AWMA)—short version, completed by the children. The BRIEF GEC T-score represents a composite of 86 items that form indices of Behavioral Regulation (includes inhibition, shifting, and emotional control) and Metacognition (includes initiative, working memory, planning/organizing, organization of material, and monitoring) [35]. The BRIEF GEC T-score reflects how children’s EF is manifested in real-world situations [35]. Lower scores represent better EF. The instrument has been shown to be reliable with an internal consistency of α = 0.80–0.98, and a test–retest reliability of *r* = 0.81 [35]. The BRIEF survey was completed by parents and graduate student research staff together. Parents dictated answers to the research staff who completed the forms and ensured that all questions were carefully considered and that no items were skipped. This process was completed 1-on-1 in a private room at the child’s school in one sitting. 

In the current study, we used two subtests of the AWMA-short version: (1) verbal working memory and (2) visuospatial working memory [36]. For the verbal test, children listened to several sentences and determined if they were true or false. Children received a point for correctly recalling the final word of each sentence in the correct order. This test has a moderate to high test–retest reliability for digit recall (0.84), word recall (0.76), and non-word recall (0.64) [36]. For the visuospatial working memory test, children looked at two shapes where the shape on the right had a red dot above it that moved in random order, while the shape itself was rotated at a different angle from its counterpart on the left. First, participants determined if the shape on the right had been rotated compared to the shape on the left. Second, they attempted to recall the position of each dot in sequence. Performance on each AWMA task is converted to an age-based standard score with distribution M = 100, SD = 15. AWMA tests have high test–retest reliability for dot matrix (0.83), maze memory (0.81), and block recall (0.83) [36]. During completion of the testing, a trained research staff member sat down with the child 1-on-1 in a quiet room at the child’s school on a day that children had not exercised. The AWMA is an automated test; hence, the computer program itself gives the instructions. The staff member oversaw administration, clarified instructions, kept children on-task, and corrected any misinterpretations of the instructions or attempts to complete the tasks quickly by hitting random buttons. 

**Academic skills** were measured with the Curriculum-Based Measurement System or CBM [37], which included three tests: (1) oral reading fluency, (2) reading comprehension, and (3) math. The oral reading fluency score was determined by asking children to read a short story aloud for 1 min, with 1 point awarded for each word read correctly. More words read correctly reflects better reading fluency. To determine the reading comprehension score, children were asked to read a grade-level passage for three minutes. Within some sentences, a parenthesis was included showing three options from which the children had to select the correct one according to the context, with each correct selection awarded 1 point. More words selected correctly reflects better reading comprehension. The math score was determined by asking children to compute several grade-level-based math problems in 3 min; 1 point was assigned for each correct digit. The higher the score obtained by the child, the better his/her math skills. Criterion validity correlations ranged from 0.70 to 0.95 [38]. During CBM testing, trained research staff sat 1-on-1 with participating children in a quiet room at the school. They read instructions for each task out loud to the student, checked for understanding, and answered any questions about what was being asked. The staff member followed along with the child during each test to record performance, keep time, score the result, and ensure children were giving full effort.

**ST and time spent in light, moderate, and vigorous PA** were collected through accelerometry. Children wore a GT3X+ or GT1M ActiGraph accelerometer on the right hip for seven consecutive days; both models collect our outcomes equivalently as we used a 60 s epoch [39]. Participants were instructed to put on the device as soon as they woke up in the morning and to remove it for aquatic activities, bathing, and before going to bed. Filters were created to remove time spent in the intervention or control condition (3:15 p.m.–6:00 p.m.). Additionally, filters were customized to each participant to remove sleeping time by calculating an average of the time they went to bed and the time they woke up (determined by consecutive zeros ranging from 5.4 to 12.1 h) based on each participant’s valid days. Families were offered USD 10 Walmart gift cards to return accelerometers. Table 1 provides a summary of the accelerometer data extraction protocol.

### 2.4. Statistical Analyses

Descriptive statistics were conducted for age, accelerometer wear time, predictor variables (ST, light PA, moderate PA, and vigorous PA), and outcome variables (BRIEF GEC T-score, AWMA-verbal and visuospatial working memory score, CBM-oral reading fluency, reading comprehension, and math scores). Bivariate correlations were conducted to test relationships between predictor and outcome variables. Linear regression analyses tested whether relationships were significant after controlling for accelerometer wear time, a conventional covariate used to control for potential differences in the time that children wore the monitors—which influences minutes of recorded PA. Assumptions for linear regression (i.e., multicollinearity, outliers, normality, linearity, homoscedasticity, and independence of errors) were checked. Variables that exceeded a kurtosis of ±3 or skewness of ±1 were transformed to achieve normality. ST and light PA were reciprocal times 1000 transformed; moderate PA was log transformed; and vigorous PA was log10 + 1 transformed. As the AWMA already controlled for age and the BRIEF GEC T-score and the Curriculum-Based Measurement System already controlled for sex and age through standardized scores, there was no need to control for these. All analyses were conducted using IBM SPSS Statistics version 24.0 [43].

## 3. Results

### Descriptive Statistics

Table 2 presents descriptive statistics for independent and dependent variables. The average wear time was 833.5 min/day, excluding intervention time. Valid accelerometer data were available for 23 children. For the CBM reading comprehension assessment, six first and second grade students were not eligible for this measure because the test is not appropriate for children below third grade. Children were low on reading fluency, reading comprehension, math proficiency, and BRIEF GEC T-score. On average, children in this sample attained few minutes of daily moderate intensity PA (under 20 min) and vigorous PA (fewer than 5 min).

**Figure 1:**
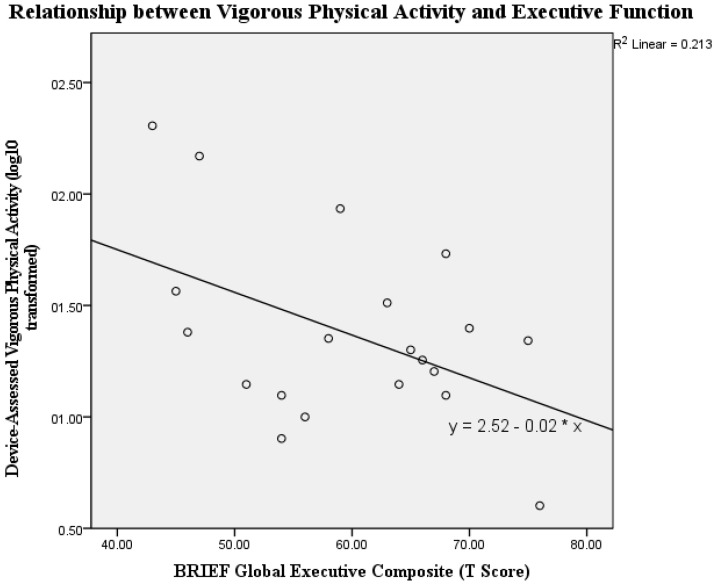
Oral reading fluency and executive functions as correlates of physical activity.

Bivariate correlations revealed that vigorous PA was associated with better BRIEF GEC T-score (r(18) = −0.46, *p* = 0.040). Table 3 summarizes the unadjusted bivariate correlations. Figure 1 illustrates the bivariate correlation between vigorous PA and BRIEF GEC T-score. This implies that more vigorous PA is related to improved EF. Wear time was not a significant covariate in the model of vigorous PA predicting BRIEF GEC T-score (*p* ≥ 0.05).

## 4. Discussion

Despite utilization of a small convenience sample, our study provides the first test of relationships between device-assessed movement behaviors, cognition, and academic performance among African American children with elevated symptoms of ADHD and DBD living in a low-income community. Our interest in testing relationships in this understudied population stemmed from questions regarding whether the additional challenges faced by children in these communities would diminish established relationships between these factors. While results from such a small study can never be considered definitive, several interesting results emerged. Namely, even though participant characteristics differed from the general population, evidence was largely consistent with the broader literature on physical activity and ADHD.

Overall, findings revealed low attainment on reading fluency, reading comprehension, math proficiency, and BRIEF GEC T-score. Additionally, low levels of PA in this sample contrast with national samples of the general population in this age group that estimate moderate to vigorous PA from accelerometers at 50 min per day [40]. However, ST was relatively low compared to national accelerometer data reported by Katzmarzyk and colleagues [44] (521 min per day vs. 295 min per day in our sample), perhaps owing to our sample selected for externalizing behavior problems. This difference in PA and ST levels might be due to the epochs selected in each study even though cutoff points were the same. In the current study, we used a 60 s epoch while Katzmarzyk et al. used a 15 s epoch. Despite this small sample, a significant correlation emerged between vigorous PA and executive function. No relationships were evident for light or moderate PA on any outcome. These findings support Gapin and Etnier [15], who reported relationships between device-based PA and EF among a sample of predominantly White children with ADHD (*n* = 18, mean age = 10.6 years, all boys); specifically, moderate to vigorous PA (both intensities combined) was positively associated with an overall measure of EF (Tower of London Total Move Score and Tower of London Total Execution Time). Light PA was not related to any cognitive outcomes in that study (consistent with our findings). ST and academic outcomes were not evaluated in that study.

No relationships were evident between ST, EF, and academic skills. The lack of relationship might be due to the way ST was measured in this study—through accelerometry rather than recreational screen time. Some sedentary activities (reading and studying) can improve EF and academic skills in children with ADHD. For instance, a systematic review that examined the relationships between self-reported and objectively measured ST and physical and mental health indicators in children reported that longer periods of reading and doing homework were related with better grade point average, standardized test scores, and reading and math skills [45]. It might be that analyzing objectively measured ST by periods of the day such as before school, during school, and after school, rather than the whole day, will help identify meaningful relationships between ST and EF or academic skills at certain periods of the day. For example, a positive relationship between ST and academic skills may be more likely during school time in which students are engaged in reading and studying. Similarly, one might expect a negative relationship of EF and academic skills with ST before and after school time.

This study has limitations. The cross-sectional design cannot be used to infer direction of influence. It is likely both that PA influences EF (as discussed above) and that EF influences PA (e.g., children with higher EF are more likely to find and engage with structured PA opportunities). The BRIEF measure adjusts for age and sex and we considered accelerometer wear time as a covariate, but there are other covariates that might influence the relationships. For example, stress may influence the relationships tested since it has been reported that stress is inversely associated with EF and moderate to vigorous PA [46]. In addition, the accelerometer models utilized limited our ST and PA data processing to using 60 s epochs, which makes it difficult to compare our data with other studies using 15 s epochs. Lastly, most students did not return their accelerometers with usable data and were excluded from analyses. Most often, the children simply lost the accelerometer, and the returned accelerometers trickled back to the research team in the ensuing months having recorded little or no data. Mostly this occurred after we offered families USD 10 gift cards for each accelerometer returned.

Despite these limitations, the study possesses multiple strengths. One strength of this study was the use of accelerometers to measure PA and ST. To our knowledge, this is the first study reporting relationships between ST and academic outcomes in children with ADHD regardless of sociodemographic backgrounds. Although our sample size was small and from a single school, findings offer fresh insight into associations among PA, ST, EF, and academic outcomes for African American children with ADHD and DBD in a disinvested urban community—a demographic that has largely been excluded from studies examining these relationships.

## 5. Conclusions

This study tested the relationships of device-measured PA and ST with EF and academic skills among African American children with elevated symptoms of ADHD and DBD in a low-income neighborhood. It used established valid measures of EF and academic skills for children and accelerometer measures of PA and ST. Despite the small sample, findings were largely consistent with our hypotheses. Light PA was not related to better EF and academic skills, which we anticipated. Moderate PA was not related to EF or academic skills. This was surprising as consistent experimental studies show relationships between PA and EF, and other cross-sectional studies of similar size have observed this relationship. Vigorous PA and EF were related, as expected; however, data did not support the relationship between vigorous PA and academic skills. Lastly, findings did not support our hypothesized inverse relationship of ST with EF and academic skills. Findings bring to our attention the diversity of ST behaviors. Our findings provide initial evidence that the additional challenges faced by African American children with ADHD and DBD in low-income communities do not overwhelm established relationships between PA and cognition. Future studies should use representative samples, longitudinal and experimental designs, and explore qualitative factors associated with different forms of PA and ST, to elucidate characteristics of PA that are related to EF and academic skills.

## Figures and Tables

**Table 1:** Accelerometer protocol to download and analyze the data.

Parameters	Rationale
Location: right hip	Most accurate place for assessing sedentary time and PA intensities [40].
Sampling Frequency: 90 Hz	
Valid Day: 8 h/day	Recommended this minimum for a valid day’s measurement [40].
Valid Week: 3 weekdays/week	Migueles et al. [40] recommended 4 days/week when analyzing 8 days’ data. However, for this paper, we will analyze data of 5 weekdays only, requiring 3 valid weekdays.
Epoch: 60 s	We used 60 s length because some children wore a GT1M ActiGraph accelerometer which does not allow analysis of data with a shorter length epoch.
Non-wear Time Validation: 20 min of consecutive zeros	Toftager et al. [41] suggested 20 min of consecutive zeros for overweight children and most of our sample was overweight.
Registration Period: Waking hour protocol	Waking hours were used to avoid sleep disruptions.
Intensity Classification: Evenson cutoff points [42]	Most accurate for the age range and is commonly used in this population [40].

**Table 2:** Descriptive statistics of the predictor and outcome variables.

Predictor Variables	*n*	Mean (SD)
Sedentary Time (min/day)	23	295.6 (67.2)
Light PA (min/day)	23	281.9 (54.9)
Moderate PA (min/day)	23	19.8 (9.5)
Vigorous PA (min/day)	23	3.7 (4.0)
**Outcome Variables**		
BRIEF GEC T-score	20	59.8 (10.0)
AWMA-verbal score	18	90.9 (13.8)
AWMA-visual spatial score	18	95.2 (11.3)
CBM-oral reading fluency score	19	68.9 (37.7)
CBM-reading comprehension score	15	9.8 (6.1)
CBM-math score	20	20.3 (7.4)

Note: SD = standard deviation, PA = physical activity, BRIEF GEC T-score = Behavior Rating Inventory of Executive Function Global Executive Composite, AWMA = Automated Working Memory Assessment Standard Score, CBM = Curriculum-Based Measurement System.

**Table 3:** Correlation matrix for predictor and outcome variables.

	Measure	1	2	3	4	5	6	7	8	9	10
1	Sedentary Time	-									
2	Light PA	−0.25	-								
3	Moderate PA	0.19	−0.37	-							
4	Vigorous PA	−0.13	−0.09	0.50 *	-						
5	BRIEF GEC T-Score	0.02	−0.11	−0.02	−0.46 *	-					
6	AWMA—Verbal Score	0.12	0.21	0.12	0.18	0.08	-				
7	AWMA—Visual Spatial Score	0.40	−0.44	0.23	0.09	−0.01	0.31	-			
8	CBM—Oral Reading Fluency Score	0.15	−0.23	0.31	0.12	−0.08	0.42	0.31	-		
9	CBM—Reading Comprehension Score	−0.09	0.05	−0.02	0.08	−0.17	0.43	0.14	0.70 *	-	
10	CBM-Math Score	0.05	−0.04	−0.06	0.20	−0.58 *	0.35	−0.03	0.33	0	-

Note: PA = physical activity, BRIEF GEC T-score = Behavior Rating Inventory of Executive Function Global Executive Composite, AWMA = Automated Working Memory Assessment, CBM = Curriculum-Based Measurement System. Sedentary Time and Light PA were reciprocal times 1000 transformed. Moderate PA was log-transformed. Vigorous PA was log 10 plus 1 transformed. * *p* < 0.05.

## Data Availability

The data presented in this study are available upon reasonable request from the corresponding author. The data are not publicly available because participants of the current study did not consent for their individual data to be shared with journal outlets.
